# Felidæ in Captivity

**Published:** 1880-07

**Authors:** Wm. A. Conklin

**Affiliations:** Director New York Central Park Menagerie; New York


### ART. XI.—HABITS AND TREATMENT OF THE FELIDÆ IN CAPTIVITY.

BY WM. A. CONKLIN,
DIRECTOR NEW YORK CENTRAL PARK MENAGERIE.

AS the Felidæ family comprises some of the most valuable and interesting animals to be found in the zoological gardens and menageries, their treatment in captivity is of great importance. It is impossible, in view of their confinement and consequently limited opportunities of exercise, for them to have all the conditions of life favorable to health. Constipation is one of the serious difficulties we have to contend with. To guard against this, liver is fed them once a week; when this proves ineffectual, castor oil is administered with their meat.

Their food is either beef or horse-flesh, the latter being used altogether in the Zoological Garden of Philadelphia, and in the principal European gardens, as a measure of economy. Old and worn-out horses are taken to the gardens, where a veterinary surgeon examines them to ascertain if they are free from disease. In some gardens, the mode of killing these horses is by a blow on the head, as this retains the blood through the meat. Regularity as to time of feeding is of importance. We feed once on week-days only, and fast on Sunday, in order to avoid surfeiting and to give the stomach a rest. The animals understand this perfectly; for while at the approach of the feeding hour during the week, they pace their cage, impatiently watching for the keeper, on Sunday, they give no sign of expectation, but remain quiet in their cage. I have had instances of animals voluntarily abstaining from food for a period of ten days without suffering apparent inconvenience. In cases of this kind, I try to tempt their appetite with a live rabbit, or chicken, or other delicate morsel. The tiger and lion are allowed for one meal twelve to fifteen pounds of meat, including bone; the smaller animals less, in proportion to their size. When the meal is finished, water is given them, after which they pace the cage for awhile, and then sleep. They are all very fond of catnip, and when it is placed in their cage, enjoy rolling in it, thus resembling the domestic cat. It is advisable to place logs of wood in the cage, so that the animal may scratch with its claws; otherwise, the nails will grow into the flesh and fester, causing much pain, and will have to be cut from time to time.

The Felidæ live in captivity from fifteen to sixteen years, showing signs of decay at about the age of twelve. The lion seems to breed more freely than any other species of the Felidæ; and strange to say, that in traveling menageries, they breed as freely as in gardens where the cages are more spacious. This may be attributed to the change of air and scene in their circus life. The male will serve the female at least thirty times a day as long as she is in season, which lasts generally ten or twelve days. The jaguar and leopard have been crossed, also the tiger and lion. Specimens of the latter were exhibited last year in this city by Mr. Forepaugh in his traveling menagerie. Period of gestation in lion, tiger and hyena sixteen weeks, leopard fifteen weeks, panther thirteen weeks. The number at a birth is usually from two to four, in the case of the lion an hour or two intervening between the delivery of each cub. The last time a hyena at the menagerie gave birth to young there was an interval of twenty-four hours between each cub. Weight of lion-cubs when born four to six pounds, hyena three and a-quarter pounds. Eyes open after from four to seven days and in three weeks teeth all cut. The young of the spotted hyena are born without spots, while those of the self-colored animals, lion and panther, are born with spots which disappear in from three to four months. The young of the tiger and leopard are marked like the adult, but of course less distinctly. It frequently happens that the mother will devour her young, but after the cubs are three or four weeks old there is no further danger. The tiger cubs are generally taken from their mother as soon as born and given to a bitch to bring up.

Our cubs at the menagerie suffer chiefly from rickets which affects them when about six or eight months old. As a remedy for this we give lime and have used the calcis phosphorus precipitate with good results. We have a lioness at the Park menagerie that has bred twenty-seven cubs in seven years and raised but one. Mr. Bartlett, in a paper read before the London Zoological Society, says: "A very extraordinary malformation or defect has frequently occurred among lions produced during the last twenty years in the Regent's Park. This imperfection consists in the roof of the mouth being opened, the palatal bones do not meet, the animal therefore is unable to suck and consequently dies. This abnormal condition has not been confined to any one pair of lions, but many lions that have bred in the garden, and not in any way related to each other, have from time to time produced these malformed young, the cause of which appears to me quite unaccountable."

In the Dublin Zoological Garden they had a lioness which presented them with fifty-four cubs, fifty of which she succeeded in raising.

Referring to the death of this animal, the report of the garden for that year says: "The closing weeks of her useful life were marked by a touching incident worthy of being recorded. The large cats, or carnivores, when in health, have no objection to the presence of rats in their cages. On the contrary, they rather welcome them as a relief to the monotony of existence, which constitutes the chief trial of a wild animal in confinement. Thus, it is a common sight to see half-a-dozen rats gnawing the bones off which the lions have dined, while the satisfied carnivores look on contentedly, giving the poor rats an occasional wink with their sleepy eyes. In illness the case is different, for the ungrateful rats begin to nibble the toes of the Lord of the Forest before his death, and add considerably to his discomfort. To save our lioness from this annoyance, we placed in her cage a fine little rat tan-terrier, who was at first received with a surly growl; but when the first rat appeared, and the lioness saw the little terrier toss him in the air, catching him with professional skill across the loins with a snap as he came down, she began to understand what the terrier was for; she coaxed him to her side, folded her paw around him, and each night the little terrier slept at the breast of the lioness, enfolded with her paws, and watching that his natural enemies did not disturb the natural rest of his mistress. The rats had a bad time during those six weeks.

These animals are very decided in their likes and dislikes; they will dart at the bars of their cage and growl at any one for whom they have an aversion. They have a very retentive memory. One at the Park menagerie saw, among a crowd of visitors, a former keeper, and though he had been absent four years, the affectionate animal at once recognized him with unmistakable signs of joy.

The care of wild animals, though attended with much anxiety and frequent disappointments, is in many respects satisfying. The opportunities for a close and correct observation of their habits are great, and no one can be long associated with them without being deeply impressed by their intelligence as well as by their amiable traits.

